# Royal Netherlands Marechaussee Personnel’s Self-Perceived Occupational Demand Profiles: A Latent Profile Analysis Shows the “Good” Versus the “Bad”

**DOI:** 10.1093/milmed/usad077

**Published:** 2023-03-25

**Authors:** Pablo M Stegerhoek, Jesse van der Zande, Caroline Bolling, Herman IJzerman, Evert A L M Verhagen, P Paul F M Kuijer

**Affiliations:** Amsterdam Collaboration on Health and Safety in Sports, Department of Public and Occupational Health, Amsterdam Movement Sciences, Amsterdam UMC, Amsterdam 1081 BT, The Netherlands; Amsterdam UMC, University of Amsterdam, Department Public and Occupational Health, Amsterdam Public Health Research Institute, Amsterdam, The Netherlands. Academic Medical Centre, Meibergdreef 9, Amsterdam 1105 AZ, The Netherlands; Amsterdam UMC, University of Amsterdam, Department Public and Occupational Health, Amsterdam Public Health Research Institute, Amsterdam, The Netherlands. Academic Medical Centre, Meibergdreef 9, Amsterdam 1105 AZ, The Netherlands; Health Care Section, Royal Netherlands Marechaussee, Plein-Kalvermarkt-Complex, Den Haag 2511 CB, The Netherlands; Amsterdam Collaboration on Health and Safety in Sports, Department of Public and Occupational Health, Amsterdam Movement Sciences, Amsterdam UMC, Amsterdam 1081 BT, The Netherlands; Health Care Section, Royal Netherlands Marechaussee, Plein-Kalvermarkt-Complex, Den Haag 2511 CB, The Netherlands; Amsterdam Collaboration on Health and Safety in Sports, Department of Public and Occupational Health, Amsterdam Movement Sciences, Amsterdam UMC, Amsterdam 1081 BT, The Netherlands; Amsterdam Collaboration on Health and Safety in Sports, Department of Public and Occupational Health, Amsterdam Movement Sciences, Amsterdam UMC, Amsterdam 1081 BT, The Netherlands; Amsterdam UMC, University of Amsterdam, Department Public and Occupational Health, Amsterdam Public Health Research Institute, Amsterdam, The Netherlands. Academic Medical Centre, Meibergdreef 9, Amsterdam 1105 AZ, The Netherlands

## Abstract

**Introduction:**

Research has linked high occupational demands to multiple adverse health outcomes, both physical and mental. As far as we know, researchers have not identified the profile characteristics of military police personnel based on occupational demands. The current study aims to identify profiles based on self-perceived occupational demands and work-related factors. This study is a starting point for characterizing performance and health in a military police population.

**Methods:**

This was a cross-sectional study in which we gathered survey data from 1,135 Royal Netherlands Marechaussee members. We used Latent Profile Analysis to identify profiles based on nine indicators of workload and work characteristics selected via focus groups and interviews with Royal Netherlands Marechaussee personnel. We determined if the profiles differed significantly across all indicators with an analysis of variance. Then, we used binominal logistic regression to determine the odds ratio (OR) for the indicators on profile membership.

**Results:**

We discovered two profiles that were distinct across all indicators. Experience (OR = 1.02, 95% CI [1.00–1.04]), autonomy (OR = 1.18, 95% CI [1.06–1.31]), task clarity (OR = 1.49, [1.32–1.69]), and work support (OR = 2.63, 95% CI [2.26–3.09]) were all predictors for a low perceived occupational demand profile. In contrast, mental (OR = 0.18, 95% CI [0.13–0.25]) and physical (OR = 0.42, 95% CI [0.32–0.54]) fatigue, and boredom (OR = 0.14, 95% CI [0.10–0.20]) were predictors for high perceived occupational demand profiles.

**Conclusion:**

We established two distinct profiles that describe the characteristics reported by the Royal Netherlands Marechaussee personnel based on workload and work characteristics. High scores on autonomy, work support, and task clarity predict favorable perceived occupational demands, whereas fatigue and boredom predict unfavorable occupational demands. Remarkably, the physical workload did not predict high perceived occupational demands.

## INTRODUCTION

Are you up to the task? Previous research has linked high occupational demands to adverse effects on health, poor well-being, and absenteeism.^1–3^ Employers can sometimes adjust work-related factors to reduce these adverse effects. In other cases, however, these demands are unmodifiable. Unmodifiable demands may be specifically present for highly demanding professions such as first responders, police officers, firefighters, and military personnel. These professions are characterized by high physical and mental demands.^4–6^ In these professions, it is essential to identify the characteristics that enable personnel to operate well regardless of the demands.^2,4^ Understanding these characteristics might help to prevent health problems, poor well-being, absenteeism, and poor performance. The International Classification of Functioning Disability and Health (ICF) model may help us understand these characteristics better.^7^ The ICF provides a framework that allows us to describe functioning within individuals in a multidimensional manner.^8^ By monitoring all components of the ICF model, we hope to facilitate a better understanding of functioning within highly demanding professions.

The Royal Netherlands Marechaussee (RNLM) describes its tasks as follows:

The Royal Netherlands Marechaussee safeguards the state’s security in The Netherlands and further afield. It is deployed globally at locations of strategic importance: from royal palaces to the external borders of Europe and from airports in The Netherlands to theatres of war and crisis areas all over the world.^9^

Their tasks can be characterized by mainly being monotonous while having the potential to be highly volatile. While guarding structures of interest, for example, most of the time is spent sitting, standing, or walking. However, personnel may be required to perform sudden intense bouts of physical or mental exertion at any time. The strain on the personnel leads to a high prevalence of dropouts or extended sick leave.^10^ In recent years, the RNLM has had an increasing range of tasks with, as a result, increasing demand for personnel. Therefore, any personnel removed from the workforce puts extra strain on the organization.

Previous research in similar populations has looked at specific risk factors for musculoskeletal injury, cardiovascular disease, and premature occupational discharge.^11–14^ Common risk factors were age, smoking, or limited physical activity for musculoskeletal injuries;^11^ hypertension, smoking, and obesity for cardiovascular disease;^13^ and injuries, little physical activity, and poor mental health for premature occupational discharge.^12^ However, these studies do not account for occupational characteristics, while these characteristics may serve as (moderators for) risk factors.^15^ Therefore, we explore in this study the characteristics of RNLM personnel with the aim of better understanding their occupational demand profiles.

## METHOD

### Study Design

This is a cross-sectional study using a survey. Our Medical Research Ethics Committee waived our study from the ethical approval process. Participants received written information on confidentiality and anonymous data management and gave informed consent. We provided no financial or other incentives to complete the questionnaire.

### Participants

The RNLM consists of three branches: (1) Staff, (2) “National Centre for Training and Expertise” (OTC), and (3) “National Tactical Command” (LTC). The staff is a group of officers that manage a division or brigade or are concerned with internal policy making. OTC is the training center, and LTC is the center for all active military personnel. We aimed to include participants from the staff, OTC, and LTC; in total, 7,658 RNLM employees received the questionnaire.

### Data Collection

We distributed the survey in September 2021 and made it available for 5 weeks on the RNLM intranet. We employed multiple strategies to augment the response rate. First, the highest-ranking commanding officer within the organization (lieutenant general) sent out an email that urged employees to fill in the questionnaire (1). Next, brigade generals brought the questionnaire to all employees’ attention (2). Finally, after 4 weeks, we sent an intern reminder (email) (3). Completing the questionnaire took approximately 10 minutes and could be done during working hours.

### Measures

#### Questionnaire

When drafting the questionnaire, we aimed to make the questions relevant to all branches and ranks of the RNLM. We based the questions on the primary roles of the units (Staff, OTC, and LTC) and the workload-related critical factors mentioned during previously performed interviews and focus groups; these data are not published. Furthermore, we aimed to include reliable, valid, and responsive items where possible. The questionnaire had four main themes: (1) General information, (2) workload, (3) work ability, and (4) work characteristics. For the present study, we used data regarding general information, workload, and work characteristics.

#### General information

We gathered demographic data like gender, age, number of kids, and data regarding employment. The data regarding employment consisted of years of employment in the military, years of employment at the RNLM, time in current position, current rank, contract hours, paternity/maternity leave, hours of paternity/maternity leave per week, overtime, hours of overtime per week, current unit, main tasks, brigade, sector/managing board, managerial position, and the number of subordinates (direct and indirect).

#### Workload

We assessed workload by asking participants to rate their perceived physical and mental workload on an adjusted Borg Rating of Perceived Exertion scale,^16^ rating from 1 (not at all) to 10 (maximal). The items used for this were as follows: “Do you find your work physically demanding?” and “Do you find your work mentally demanding?.”

#### Work characteristics

Next, we surveyed the characteristics of different job aspects. We based these items on the Experience and Evaluation of Work questionnaire (Dutch: VBBA).^17^ The VBBA is reliable and unidimensional on all its scales.^17^ We asked participants, “How often do you perform the following activity during your typical workday?”. We asked this question for sitting, standing, driving, and walking. The answer categories ranged from 1 “rarely or never” to 5 “(almost) always.” Boredom and physical and mental fatigue had a single item with the same answer options (1–5). We used two items for job autonomy, task clarity, and work support. These items were as follows: “I can do my job according to my understanding” and “I can plan my job according to my own understanding” for autonomy, “Do you know exactly which task you are and aren’t responsible for?” and “Do you know exactly what your task is?” for task clarity, and “I experience support from colleagues during work” and “I experience support from my superior during work” for work support. We combined the two items of autonomy, task clarity, and work support, which were scored from 1 to 5 to result in a summarized score of 1–10 for autonomy, task clarity, and work support. Finally, we asked participants about body armor, shift work, and overtime.

### Statistical Analysis

#### Descriptive analysis

First, we described the characteristics of the participants overall and per unit using IBM SPSS statistics version 28 (IBM Corp.) (Table I). We compared our sample’s gender, unit, and function to the entire RNLM to check our sample’s representativeness (Table II).

**TABLE I.** Descriptive Data for All Units

		LTC		OTC		Staff		Total	
		(*n* = 1,135)		(*n* = 182)		(*n* = 221)		(*n* = 1,538)	
		*n*	%	*n*	%	*n*	%	*n*	%
Sex	Male	909	80	133	73	136	62	1178	77
	Female	217	19	42	23	82	37	341	22
	Transgender	2	0	1	1	0	0	3	0
	Prefer not to say	7	1	6	3	3	1	16	1
Age category (years)	≤29	257	23	33	18	20	9	307	20
	30–39	335	30	45	25	46	21	426	28
	40–49	261	23	48	26	55	25	364	24
	>50	282	25	56	31	100	45	438	28
Children	Yes	702	62	119	65	145	66	966	63
	No	433	38	63	35	76	34	572	37
Type of employee	Military	1000	88	156	86	100	45	1256	82
	Civilian	125	11	25	14	117	53	267	17
	Reservist	10	1	1	1	4	2	15	1
Rank	Marechaussee	76	8	22	14	3	3	101	8
	Petty officer	711	70	88	56	15	14	814	64
	Officer	216	21	44	28	77	74	337	26
	Chief officer	8	1	3	2	9	9	20	2
	No rank/not known	124	11	25	14	117	54	266	17
Overtime	Yes	766	68	79	43	135	62	980	64
	No	369	33	103	57	84	38	556	36
Managerial position	Yes	449	40	64	35	42	19	555	36
	No	686	60	118	65	177	81	981	64
Body armor	Yes	438	43	15	10	1	1	454	36
	No	572	57	142	90	102	99	816	64
Irregular shifts	Yes	526	46	9	5	4	2	539	35
	No	609	54	173	95	215	98	997	65

**TABLE II.** The Demographics of All Employees of the RNLM in Comparison to the Demographics of the Present Study’s Participants

	RNLM	% RNLM	Included	% Included	Difference
Total	7,658		1,538		
Male	6,112	80	1,178	77	3
Female	1,546	20	341	22	2
Different/unknown	–	–	19	1	–
Military	6,606	86	1,256	82	4
Civilian	746	10	257	17	7
Reservist	306	4	15	1	3

#### Latent Profile Analysis

We used R,^18^ with the mclust^19^ package, to perform a Latent Profile Analysis (LPA). For the LPA, we selected nine indicators based on statistical appropriateness, interpretability, and theoretical support (Table S1).^20^ We made this selection in collaboration with the RNLM through focus groups and interviews in a preceding qualitative part of this project. This selection consisted of four variables relating to occupational demands: physical workload, mental workload, physical fatigue, and mental fatigue. Experience, boredom, autonomy, work support, and task clarity are indicators of work characteristics.

Latent Profile Analysis is a probabilistic approach that distributes data in *k* clusters where *k* is not known *a priori*. There are multiple methods and criteria to estimate the appropriate number of clusters. First, we looked at the Integrated Complete Likelihood (ICL) and the Bayesian Information Criterion (BIC),^21^ where lower values constitute a better fit. Next, we used the Bootstrap Likelihood Ratio^22^ to examine if a model with *k* clusters was a better fit to the data than a model with *K-1* clusters (*P* < .05). Finally, when deciding on the final model, we aimed to facilitate interpretability and practical relevance.^23,24^ We displayed the outcome in the number of SDs from the mean using *z*-scores. Next, we assessed differences between the profiles in terms of the indicators with univariate analysis of variance. Finally, we used a binary logistic regression model to determine the odds ratio (OR) of profile membership for each previously selected indicator.

## RESULTS

### Descriptive Characteristics

At the time of the inclusion, the RNLM had 7,658 employees, of which 1,763 completed the survey. Two hundred and twenty-five were not submitted and had missing items or unreliable data; we removed these items. We performed the final analyses on the data of 1,538 respondents (response rate: 23%). Of these 1,538 respondents, LTC employed 1,135 (74%) personnel, 221 (14%) were staff, and 182 (12%) worked at the OTC. Our sample’s demographics were comparable to the RNLM population, except for a higher percentage of civilians in our sample (7%) (Table II). Our sample consisted of 1,178 (77%) males, 341 (22%) females, 3 (0%) transgender, and 16 (1%) who chose not to specify. Of these, 307 (20%) were 29 years of age or younger, 426 (28%) between 30 and 39 years, 364 (24%) between 40 and 49 years, and 438 (28%) were 50 years or older.

The mean physical workload was 4.0 (SD 2.6), and the average mental workload was 6.7 (SD 2.5). Seventy-five percent of the respondents reported physical fatigue, while 93% reported mental fatigue.

Thirteen hundred fifty-six employees (88%) said they were never or rarely bored. The average scores for task clarity, autonomy, and work support were as follows: 7.8 (SD 1.9), 7.5 (SD 2.0), and 7.6 (SD 1.9), respectively.

### Latent Profile Analysis

**FIGURE 1.**
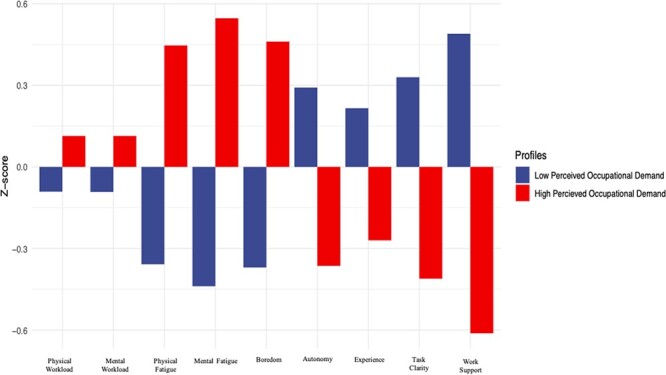
The two self-perceived occupational demand profiles by the Latent Profile Analysis (LANDSCAPE).

BIC and ICL indicated that an ellipsoidal, equal volume, and equal shape, with six profiles, was optimal for our data. The Bootstrap Likelihood Ratio showed significant *P*-values for up to at least six clusters (*P* < .05). However, six clusters would make the model difficult to interpret. The BIC and ICL values were reasonably constant for all models from one to five clusters. The ellipsoidal, equal shape, and orientation (VEE) two-cluster model yielded the best BIC, and ICL resulted in this range. Given that the practical applicability increased substantially from a five- to a two-cluster model, we selected a VEE model with two clusters. The outcome of the LPA can be seen in Figure 1. We named the clusters“high perceived occupational demand” (HPOD) and “low perceived occupational demand” (LPOD), and as seen in Figure 1, they represent distinct patterns across the indicators. When occupational demand indicators are high, autonomy, experience, task clarity, and work support are low and vice versa.

Table III shows the analysis of variance results for comparing the two profiles across all indicators and the regression results for predicting profile membership across all indicators. All indicators, physical workload (*F*(1,1214) = 26.48, *P* < .001), mental workload (*F*(1,1214) = 28.29, *P* < .001), physical fatigue (*F*(1,1214) = 24.90, *P* < .001), mental fatigue (*F*(1,1214) = 99.67, *P* < .001), and boredom (*F*(1,1214) = 149.88, *P* < .001), were all higher for the HPOD profile compared to the LPOD profile. However, experience (*F*(1,1214) = 10.94, *P* < .001), autonomy (*F*(1,1214) = 292.79, *P* < .001), task clarity (*F*(1,1214) = 65.47, *P* < .001), and work support (*F*(1,1214) = 2331.26, *P* < .001) were significantly higher for the LPOD profile compared to the HPOD profile.

**TABLE III.** Analysis of Variance Results Comparing Profiles 1 and 2 Across the Eight Indicators and Binominal Logistic Regression Results to Predict Profile Membership

	LPOD (*n* = 439), mean (SD)	HPOD (*n* = 777), mean (SD)	*F* (1,1214)	HPOD vs LPOD, odds ratio (95% CI)
Experience (years)	18.34 (11.51)	16.15 (10.32)	10.94***	1.02 (1.00–1.04)*
Physical workload (1–10)	4.33 (2.71)	3.54 (2.35)	26.48***	0.96 (0.88–1.05)
Mental workload (1–10)	6.14 (2.58)	7.67 (2.08)	114.16***	1.41 (1.25–1.59)**
Physical fatigue (1–5)	2.36 (1.06)	2.11 (0.95)	15.97***	0.42 (0.32–0.54)**
Mental fatigue (1–5)	2.56 (0.99)	3.65 (0.58)	368.44***	0.18 (0.13–0.25)**
Boredom (1–5)	1.26 (0.47)	2.03 (1.16)	149.88***	0.14 (0.10–0.20)**
Task clarity (1–10)	8.68 (1.20)	6.29 (2.02)	673.92***	1.49 (1.32–1.69)**
Autonomy (1–10)	7.71 (1.94)	7.18 (2.16)	19.30***	1.18 (1.06–1.31)**
Work support (1–10)	8.27 (1.50)	6.49 (1.95)	316.72***	2.63 (2.26–3.09)**

*F*: effect size (degrees of freedom),

* *P* < .05,

** *P* < .01,

*** *P* < .001.

All indicators except physical workload (OR = 0.96, 95% CI [0.88–1.05]) were significant predictors of profile membership. Experience (OR = 1.02, 95% CI [1.00–1.04]), mental workload (OR = 1.41, 95% CI [1.25–1.59]), task clarity (OR = 1.49, [1.32–1.69]), autonomy (OR = 1.18, 95% CI [1.06–1.31]), and work support (OR = 2.63, 95% CI [2.26–3.09]) were associated with the LPOD profile. In contrast, physical (OR = 0.42, 95% CI [0.32–0.54]) and mental (OR = 0.18, 95% CI [0.13–0.25]) fatigue and boredom (OR = 0.14, 95% CI [0.10–0.20]) were associated with the HPOD profile (Table III).

## DISCUSSION

### In Line with the Research

Using an LPA, we identified an HPOD and an LPOD profile among RNLM personnel. The HPOD or the “bad” profile describes employees who perceive their workload and fatigue as high but autonomy, experience, work support, and task clarity as low. The LPOD or “good” profile describes the opposite pattern. Previous literature indicates that autonomy, task clarity, experience, and work support moderate the occupational demands–strain relationship.^10,25–29^ This is in line with the current study, where high occupational demands were related to low autonomy, task clarity, experience, and work support.

Remarkably, the perceived physical workload was not a predictor of either profile. We can explain this by the fact that at the RNLM, and in the military in general, the emphasis is on the physical aspect.^11^ The recruits undergo intense physical training and must maintain a certain degree of fitness throughout their career.^4^ As a result, personnel are likely to be physically well trained and, hence, the low scores for the perceived physical workload. Moreover, some of the personnel do not have physically demanding tasks.

Contrary to what we found in previous studies, the high mental workload was a predictor for the LPOD profile.^30,31^ We can explain these findings through the nature of the RNLM’s tasks. Some of the typical tasks are not very physically or mentally challenging; they are monotonous (i.e., building surveillance). This could result in a higher mental workload because of boredom, positively influencing the perceived occupational demands. Abazari and colleagues link mental workload to boredom, which in our study was the strongest predictor for the HPOD profile. They found that a high mental workload, precisely the performance dimension, is associated with boredom.^32^ Our study confirms these findings.

Work support was the strongest predictor for the LPOD profile. The importance of social support in military populations, especially by superiors, has been highlighted in previous research.^33,34^ Woo et al. demonstrated that personnel who received appraisal and emotional support from superiors were less prone to depression. Interestingly, Woo et al. found no link between informational and instrumental support and depression.^34^

Finally, we established indicators in three components of the ICF model for participation. Physical and mental fatigue are “body functions and structures”; workload, task clarity, experience, autonomy, and work support are “environmental factors”; and experience is a “personal factor”.^7,8^ With this, we show that most factors contributing to the perception of occupational demands are contextual and not related to the activity itself. Additionally, we show that our indicators give a complete and multidimensional view of the functioning within the RNLM.

### Practical Relevance

These results potentially provide new, practically relevant insights for military and police organizations. First, we show that mental characteristics impact our sample’s perception of occupational demands more than physical characteristics. This may not be in line with standard practice in military environments, where training is heavily focused on the physical aspects. More time for the mental aspects of the military and police occupations in training could be beneficial.

Second, the mental workload seems to be a positive factor regarding occupational demands in the current sample. Mental workload is a concept that intuitively is easy to understand but seems hard to define.^35^ We commonly accept that when the mental workload is too high, the ability to complete the task is diminished. At the same time, a low mental workload can lead to lesser performance.^35^ This implies a “sweet spot” for the mental workload. Abazari and colleagues link mental workload to boredom and show that the performance dimension of mental workload and boredom have an inverse relationship.^32^ This concurs with the previous statement regarding low mental workload. In addition, boredom was the main predictor for the HPOD profile. Bartone et al. described boredom as one of the five main psychological stressors in the modern military.^36^ Based on the literature and the current research, we posit a possible moderating role for the mental workload on boredom in military and police personnel. Therefore, modifying the mental workload, or factors relating to mental workload, for personnel may be interesting to military organizations since boredom is one of the main stressors.

Third, the discovery of two distinct profiles based on the perception of occupational demands indicates that personal and occupational characteristics can serve as moderators for the perception of occupational demands. Autonomy, work support, and task clarity are all factors that employers can potentially modify, thereby enhancing personnel’s ability to handle occupational demands.

Another interesting avenue to explore is work support. In the present study, work support was the most significant predictor for the LPOD profile. To comprise the work support item, we combined two separate questionnaire items. One item focused on colleagues’ support, and the other one was on superiors’ support. While support from colleagues may be challenging to adjust for organizations, superior support is relatively easy to modify in the strict hierarchical military environment.^34^

### Strengths and Limitations

This study has two main strengths. First, a large group with relatively similar characteristics to the RNLM population completed the survey. Second, using an LPA enabled us to discover latent profiles based on occupational demands.

However, our study is not without its limitations. First, the response rate to our questionnaire was low compared to other studies using occupational surveys.^37^ While the characteristics of our sample were mostly like those of the entire RNLM population, we did include a higher percentage of civilians. The RNLM context may also be different from the military police context in other countries. Therefore, there is a risk of bias, and caution should be taken when extending these results to a broader military population. Next, since we analyzed cross-sectional data, no causal inferences can be made regarding our findings. Finally, an LPA is not without its limitations. There is no consensus yet on the best way to determine the number of profiles. Future developments in the analysis should provide more insights into this methodological aspect.

### Future Directions

Future research should verify these latent profiles in other military or police populations. From there, future studies should further examine the relationship between mental workload and boredom. Intervention studies that aim to increase mental workload and investigate its effect on boredom could also be valuable. Finally, more research is needed to establish the social support personnel receive from superiors. Researchers may also explore if superiors are able and willing to provide more emotional and appraisal support to their inferiors.

## CONCLUSION

We established two distinct occupational demand profiles among RNLM personnel: the HPOD or “bad” profile and the LPOD or “good” profile. The strongest predictors for the LPOD profile were mental workload, task clarity, autonomy, and work support. Physical and mental fatigue and boredom were the strongest predictors for the HPOD profile. Interestingly, the physical workload was not a strong predictor for the HPOD profile.

## Supplementary Material

usad077_Supp

## Data Availability

These data cannot be shared publicly because of the participants’ privacy. The data will be shared on reasonable request with the corresponding author.

## INSTITUTIONAL CLEARANCE

Does not apply.
