# Supplementary material for: Royal Netherlands Marechaussee Personnel’s Self-Perceived Occupational Demand Profiles: A Latent Profile Analysis Shows the “Good” Versus the “Bad”
Source: Mil Med. 2023 Mar 25;188(11-12):e3575–82. doi: 10.1093/milmed/usad077 (PMC10629987; doi:10.1093/milmed/usad077)
Supplement: usad077_Supp [file usad077_supp.zip › Table S1.docx]

| Table 1: selected items for the Latent Profile Analysis |  |
| --- | --- |
| *Items* | *Options* |
| *How long have you been working for the RNLM* | Years |
| *Do you find your work to be physically demanding?* | 1 - 10 |
| *Do you find your work to be mentally demanding?* | 1 - 10 |
| *How often are you physically tired after a workday?* | 1 - 5 |
| *How often are you mentally tired after a workday?* | 1 - 5 |
| *Are you ever bored at work?* | 1 - 5 |
| *Task clarity, the sum of 2 items (2-10):* |  |
| *Do you know, exactly, what are and aren't your responsibilities* | 1 - 5 |
| *Do you know precisely what your task is?* | 1 - 5 |
| *Autonomy, the sum of 2 items (2-10):* |  |
| *I can do my work how I think its best* | 1 - 5 |
| *I can share my work when I want* | 1 - 5 |
| *Work support, the sum of 2 items (2-10):* |  |
| *I experience support from my colleagues during my work 1 - 5* | |
| *I experience support from my manager during my work* | 1 - 5 |
| Since the original questionnaire was in Dutch, the items were translated. Years, hours, and minutes were asked in whole numbers. "->": Rate between 1 and 5 or 1 and 10. On a 10-point scale, 1 represents "not at all", and 10 means "maximal". On a 5-point scale, 1 represents never or rarely, and 5 represents (almost) always. | |
